# Super-Antioxidant Vitamin A Derivatives with Improved Stability and Efficacy Using Skin-Permeable Chitosan Nanocapsules

**DOI:** 10.3390/antiox12111913

**Published:** 2023-10-26

**Authors:** Hyeryeon Oh, Jin Sil Lee, Sunghyun Kim, Jeung-Hoon Lee, Yong Chul Shin, Won Il Choi

**Affiliations:** 1 Center for Bio-Healthcare Materials, Bio-Convergence Materials R&D Division, Korea Institute of Ceramic Engineering and Technology, 202, Osongsaengmyeong 1-ro, Cheongju 28160, Republic of Korea; hyeryeon.oh@kicet.re.kr (H.O.); jslee92@kicet.re.kr (J.S.L.); shkim0519@kicet.re.kr (S.K.); 2 School of Materials Science and Engineering, Gwangju Institute of Science and Technology, 123, Cheomdan-gwagiro, Gwangju 61005, Republic of Korea; 3 SKINMED Co., Ltd., Daejeon 34028, Republic of Korea; jhoon@cnu.ac.kr (J.-H.L.); ycshin@amicogen.com (Y.C.S.); 4 Amicogen Inc., 64 Dongburo, 1259, Jinju 52621, Republic of Korea

**Keywords:** chitosan, nanocapsule, retinyl palmitate, transdermal delivery, antioxidant, wound healing

## Abstract

Retinyl palmitate (RP) is a retinol ester with strong antioxidant and anti-inflammatory properties as an antiwrinkle agent. However, it has poor aqueous solubility and easily degrades into inactive forms for topical applications. Therefore, we developed chitosan-coated nanocapsules (ChiNCs) to encapsulate RP using a simple nanoprecipitation method for protection against physiological conditions and to enable deep skin penetration. The as-prepared RP-loaded nanocapsules (RP@ChiNCs) loaded with approximately 5 wt.% RP exhibited a hydrodynamic diameter of 86 nm and surface charge of 24 mV. They had adequate stability to maintain their physicochemical properties after lyophilization in a biological buffer. Notably, ChiNCs provided RP with remarkable protection against degradation for 4 weeks at 37 °C. Thus, RP@ChiNCs exhibited good antioxidant activity in situ for sufficiently long periods without considerable changes in their efficacy. Furthermore, ChiNCs enhanced the skin penetration of lipophilic RP based on the inherent nature of chitosan. RP@ChiNCs exhibited good in vitro antioxidant and anti-inflammatory effects without causing any cytotoxicity in dermal fibroblasts. Accordingly, they promoted cell proliferation in a wound-scratch test and enhanced collagen synthesis. These results suggest that RP@ChiNCs are promising candidates for cosmetic and biomedical applications.

## 1. Introduction

Retinol belongs to the fat-soluble vitamin family and induces the production and maintenance of mucous membranes, strong skin, and hair [1]. It is considered a potent candidate for dermatological applications; however, it exhibits lipophilicity and causes side effects such as itching, erythema, pruritus, and burning [2,3]. Moreover, it is highly susceptible to degradation in response to stimuli such as oxidation, ultraviolet radiation, heat, and moisture. The byproducts of degraded retinol may damage proteins, lipids, nuclear DNA, or cell membranes, which may cause undesired inflammation, cardiovascular diseases, and aging [4,5,6,7]. Retinyl palmitate (RP) is an ester derivative of retinol with better chemical stability. It is thought to be less susceptible to oxidation and isomerization than retinol [4], and it exerts a beneficial effect on skin after hydrolyzation to retinol via esterase enzymatic cleavage. It has been exploited as a potent therapeutic based on its antioxidant, anti-inflammatory, and antimicrobial activities. Tao et al. revealed that RP suppressed the activation of Bax and caspase-3 via regulation of the balance between intracellular oxidants and antioxidants. As a result, it could ameliorate myocardial ischemia/reperfusion injury [8]. RP also exhibit a beneficial effect on acne owing to its antimicrobial activity and anti-inflammatory effect [9]. In particular, the retinol ester RP is affiliated with the endogenous retinoid family, which decreases roughness, improves elasticity of the skin, forms healthy and uniform skin, and blocks skin lipid peroxidation by exfoliation [1]. In addition, studies have reported that RP boosts moisturization and alleviates wrinkles; thus, it is commonly used in topical creams or gels based on its antioxidant efficacy and control over the loss of collagen through skin atrophy inhibition [10,11]. Furthermore, RP is widely employed in antiwrinkle formulations as it promotes the densification of the epidermis and the efficient treatment of skin defects [12,13,14].

Although RP has potent cosmeceutical advantages, it has low efficacy and unwanted secondary effects. Importantly, even RP, the most stable retinol derivative, faces several obstacles such as hydrophobicity, toxicity at high accumulation, instability, and the possibility of increasing skin sensitivity. In the study of Bitarafan et al., RP did not significantly affect gene expression related to the cytokines involved in inflammation, whereas its anti-inflammatory efficacy has been reported previously [15]. This indicates the requirement of an effective delivery system for the protection and enhanced cellular uptake of RP. Furthermore, the regulated release of RP is important as it may disrupt the cell cycle, causing excessive oxidative stress, mitochondrial dysfunction, and apoptosis at high doses [16]. Accordingly, a novel strategy is necessary to bypass the hurdles associated with the application of RP and promote optimal RP activity. Therefore, various lipid-based encapsulation systems consisting of nanostructured lipid carriers, solid lipid nanoparticles (SLNs), nanoemulsions, and nanocapsules (NCs) have been investigated to prevent degradation, enhance aqueous solubility, and deliver active agents deep into the skin.

One of the diverse lipid-based encapsulation systems, the lipophilic particulate carrier SLN, was investigated to formulate RP [17]. Although SLN can encapsulate RP with a high loading efficiency (LE), it cannot inhibit the degradation of RP. Thus, the study suggested modifying the surface of the SLN to provide better protection against RP degradation [17]. Jeon et al. developed surface-modified lipid nanoparticles with dicetyl phosphate (DCP_mod_-SLNs) via a hot-melt technique using Gelucire50/13^®^ and Precirol AT05^®^. Consequently, negatively charged SLNs were prepared with well-distributed RP. The DCP_mod_-SLNs were uniformly prepared with sizes below 100 nm and exhibited good colloidal stability with a high zeta potential absolute value. In addition, DCP_mod_-SLNs with a negative charge improved the skin distribution of RP by approximately 4.8 times compared to SLNs, which had a neutral charge. Furthermore, DCP_mod_-SLN induced a better antiwrinkle effect than the negative control in vivo and inhibited both wrinkle formation through the low expression of elastic fibers and superoxide dismutase activity caused by UV irradiation. Nevertheless, chemical conjugation can cause unexpected side effects, high scale-up manufacturing costs, and complex steps. In addition, chemical conjugation involves multiple steps, including end-group modification and attachment of a cross-linker, which complicate the nanoparticle preparation. Importantly, it can damage biomolecules via the unwanted generation of reactive oxygen species (ROS) [18]. Therefore, a new biopolymeric carrier system that integrates stabilized antioxidants is required.

Pluronic is an amphiphilic block copolymer that self-assembles in aqueous solution due to thermodynamic incompatibility between its hydrophilic poly(ethylene oxide) and hydrophobic poly(propylene oxide) moieties [19]. Its micellar structure, containing a hydrophobic core and hydrophilic corona, provides hydrophobic molecules with better solubility and stability. In particular, RP can be encapsulated in Pluronic NCs (PluNCs) via a nanoprecipitation method. RP diffuses into the hydrophobic core of the PluNCs while the hydrophilic shell protects the drug from aggregation and degradation. PluNCs can be prepared by being coated with another polymer; thus, they exhibit better stability when exposed to varying conditions. In the biopolymer group, chitosan is nontoxic, biocompatible, biodegradable, antioxidant, and bioadhesive [20]. It is a cationic polysaccharide obtained by the deacetylation (DAC) of chitin. The interaction between chitosan and PluNC is mainly derived by electrostatic interaction and hydrogen bonding [21,22]. The positive charge of chitosan promotes an electrostatic interaction with PluNCs, leading to the formation of a chitosan layer on the PluNCs. Further, hydrogen bonding between the amine groups of chitosan and the oxygen and hydroxyl groups of the Pluronic chain has been found, as well as electrostatic interaction. Therefore, it improves pharmacokinetics and facilitates the skin penetration of NCs. It can interact with many different bioactive molecules in the presence of an amino group at C2 or hydroxyl groups at C6 of its monomeric units [23]. Chitosan itself exerts strong antioxidant properties and antibacterial activity. Furthermore, it possesses excellent advantages in the protection, controlled release, and enhanced uptake of loaded drugs [24]. Based on such synergistic relations between RP and chitosan, Fernández-Gutiérrez et al. prepared RP-loaded chitosan nanoparticles by ionic crosslinking [18]. However, RP was chemically conjugated with chitosan polymer chains using tripolyphosphate instead of being encapsulated inside NCs. 

Therefore, in this study, we developed chitosan-coated nanocapsules (ChiNCs) for the effective epidermal delivery of RP and synergistic antioxidant effects. Water-soluble chitosan with optimized DAC and molecular weight (MW) was used to prepare ChiNCs, as reported in our previous study [20]. Different amounts of RP were loaded into ChiNCs, and the physicochemical characteristics of the RP-loaded, chitosan-coated nanocapsules (RP@ChiNC) were assessed by dynamic light scattering (DLS) analysis and transmission electron microscopy (TEM). The stability of RP@ChiNCs was monitored for 4 weeks under physiological conditions. The pH and in vitro skin permeability of RP@ChiNCs were evaluated for topical application. High-performance liquid chromatography (HPLC) was used to analyze the remaining amount of RP inside RP@ChiNCs without degrading after 4 weeks of storage at 37 °C. The antioxidant activity was assessed both in situ and in vitro. Furthermore, in vitro wound healing and collagen synthesis were evaluated after RP@ChiNC treatment to determine its potential dermatological applications.

## 2. Materials and Methods

### 2.1. Materials

Water-soluble chitosan (DAC 90%, 10 kDa) was purchased from Amicogen (Jinju, Republic of Korea). Pluronic F127 (PF127; P2443), RP (R1512), acetone (650501), 2,2-diphenyl 1-1-picrylhydrazyl (DPPH; D9132), dimethyl sulfoxide (DMSO; D8418), and lipopolysaccharides from *E. coli* O111:B4 (LPS; 437627) were purchased from Sigma-Aldrich (St. Louis, MO, USA). HyClone deionized (DI) water (SH30538.03) and phosphate-buffered saline (PBS; SH30255.02) were purchased from GE Healthcare Life Sciences (Marlborough, MA, USA). Acetonitrile (HPLC grade, AH015) was purchased from Honeywell (Charlotte, NC, USA). H_2_O_2_ (30%, 23150-0350) and 2′,7′-dichlorodihydrofluorescein diacetate (H_2_DCFDA; D399) were purchased from Junsei Chemical Co. (Tokyo, Japan) and Invitrogen (Carlsbad, CA, USA), respectively. Griess reagent (ab234044) was purchased from Abcam (Cambridge, UK).

### 2.2. Preparation and Characterization of RP@ChiNCs

ChiNC-encapsulated RP with varying loading contents (LCs) was prepared as described in our previous study [20]. Nanoprecipitation was used to encapsulate hydrophobic RP into PluNCs. Briefly, 20 mg PF127 and different amounts of RP (0.4, 1, and 2 mg) were dissolved in 1 mL acetone and reacted for 2 h using a rotary shaker. The reaction mixture was then slowly added dropwise to 4 mL of DI water with stirring at 400 rpm. Magnetic stirring was continued for 12 h in a fume hood to remove the acetone. Water-soluble chitosan (20 mg) was then added to an aqueous solution of RP-loaded Pluronic nanocapsules, resulting in RP@ChiNCs after 2 h. The unloaded RP was filtered using an Amicon Ultra-15 centrifugal filter (MW cutoff, 100 kDa; Merck Millipore, Billerica, MA, USA). Bare ChiNCs were prepared using the same method with an RP LC of 0 wt.%. As-developed RP@ChiNCs were lyophilized for 3 days and stored at −20 °C until use. Briefly, they were initially frozen in a liquid nitrogen. Then, water from a frozen sample was removed by sublimation and desorption at −80 °C under a vacuum using a freeze dryer (FDU-8606, OPERON, Kimpo, Republic of Korea).

The physicochemical properties of RP@ChiNCs were characterized using several analytical techniques. The amount of unloaded RP was measured using HPLC (Waters 2695, alliance, MO, USA) to calculate the LC and LE of RP@ChiNCs. A 50 μL sample in the mobile phase (70% acetonitrile) was injected and passed through a C18 column (5 μm, 4.6 × 150 mm^2^, SunFire^®^ C18 column, Waters, MO, USA) for 20 min at 30 °C with a flow rate of 1 mL/min for the detection of RP at 325 nm. The hydrodynamic diameter, polydispersity index (PDI), and zeta potential of RP@ChiNCs with different LCs were measured using a Zetasizer instrument (ELSZ-2000, Otsuka, Osaka, Japan). The morphologies of the bare ChiNCs and RP@ChiNCs (5 wt.%) were observed using TEM (JEM-2100Plus HR, JEOL, Tokyo, Japan).

### 2.3. Stability of RP@ChiNCs

The RP@ChiNCs were lyophilized to store them in a powdered state without any changes in their properties before use. We observed the redispersing of the powdered form of the NCs in aqueous solution. The hydrodynamic diameters and PDI of RP@ChiNCs (5 wt.%) were measured before and after lyophilization. Lyophilized RP@ChiNC (5 wt.%) was redispersed in DI water and PBS for measurement. Further, NCs in aqueous solution and biological buffer were stored for 4 weeks at 37 °C and 100 rpm. At each predetermined time point (0, 1, 2, and 4 weeks), the sizes and PDI of RP@ChiNCs were measured using a Zetasizer instrument.

### 2.4. In Situ Antioxidant Activity of RP@ChiNCs

To determine the relative effect of RP stability on its antioxidant activity, samples including RP, ChiNCs, and RP@ChiNCs (2.5 mg/mL) were incubated for 4 weeks at 37 °C. Every week, the antioxidant activity was analyzed using a DPPH radical scavenging assay. Equal volumes of methanolic DPPH solution (0.5 mM) and sample solution were allowed to react for 3 h in the dark. DI water was mixed with the DPPH solution in the control group. The UV-vis absorbance was measured at 515 nm using a microplate reader (VICTOR X3; PerkinElmer, Waltham, MA, USA). The antioxidant activities of the 12 different types of chitosan were analyzed using a similar method. Considering that the antioxidant activity of RP@ChiNCs is dependent on the stability of RP, the remaining RP content in RP@ChiNCs after storage for 4 weeks at 37 °C was analyzed using HPLC as described above.

### 2.5. In Vitro Skin Permeation of RP@ChiNCs

First, the pH of RP@ChiNCs was measured using a pH meter (METTLER TOLEDO, Greifensee, Switzerland) to evaluate its suitability for topical application. The skin permeabilities of bare RP and RP@ChiNCs were compared using a Franz-type diffusion cell with full-thickness human cadaver skin samples (58 years old, male, back, certified intact skin) purchased from Hans Biomed Co. (Daejeon, Republic of Korea) and stored at −20 °C until use, as previously reported [20,25]. Skin resistivity was confirmed to be above 30 KΩ cm^2^ prior to the analysis as an indication of skin integrity. The receptor chamber was filled with 5.5 mL of PBS containing 0.5% polysorbate 80, while the donor chamber was filled with 0.2 mL of the sample solution (200 μg/mL RP). The bare RP or RP@ChiNCs were allowed to travel through the stratum corneum for 24 h at 37 °C and 100 rpm. The receptor solution (0.5 mL) was collected and replaced with fresh buffer at different time intervals to maintain the total volume. The amount of skin-permeated drug was analyzed by HPLC as described above.

### 2.6. Cell Culture

NIH 3T3 mouse normal fibroblast cells and RAW264.7 macrophage cells, purchased from Korean Cell Line Bank (Seoul, Republic of Korea), were cultured at 37 °C and CO_2_ in 10% fetal bovine serum (FBS; 16000-044, Gibco, Grand Island, NY, USA) and 1% antibiotic–antimycotic (10240-062, Thermo Fisher Scientific, Waltham, MA, USA) supplemented with Dulbecco’s modified Eagle’s medium (DMEM; 11995-065, Gibco). Human skin fibroblast samples were obtained from adult foreskins and cultured in DMEM supplemented with 10% FBS.

### 2.7. In Vitro Cytotoxicity of RP@ChiNCs

The biocompatibility of RP@ChiNCs was evaluated in NIH 3T3 fibroblasts. A Cell Counting Kit-8 (CCK8; CK04, Dojindo Laboratories, Kumamoto, Japan) was used as described previously [26]. Fibroblasts were seeded in a 96-well plate at a density of 10,000 cells/well. After 12 h of incubation, the cells were treated with different concentrations of RP@ChiNCs, from 0.01 to 1 mg/mL. After 24 h of treatment, the CCK8 solution diluted to 10% in DMEM was added to the cells for 30 min, and the absorbance at 450 nm was measured using a microplate reader. The cell viability of the control group was considered 100%, whereas that of RP@ChiNCs was evaluated as a relative percentage of the control.

Similarly, the viability of human dermal fibroblasts after treatment with RP or RP@ChiNCs (0.25%) was analyzed using 3-(4,5-dimethythiazol-2-yl)-2,5-diphenyl tetrazolium (MTT; M6494, Invitrogen, Carlsbad, CA, USA). Cells were seeded in a 48-well plate at a concentration of 2 × 10^4^ cells/mL and incubated for 24 h in 5% CO_2_ [27]. Then, MTT solution (1 μg/mL) was added to the cells for 3 h at 37 °C. After the reaction, the formazan from viable cells was dissolved using 100 μL DMSO, and then a microplate reader was used to measure absorbance at 540 nm.

### 2.8. In Vitro Antioxidant and Anti-Inflammatory Activities of RP@ChiNCs

The in vitro antioxidant activity of RP@ChiNCs was evaluated using H_2_O_2_-stimulated NIH 3T3 cells. DMEM containing 10% FBS was used as the cell culture medium. The cells were seeded in a 96-well plate at a density of 10,000 cells/well and incubated for 12 h at 37 °C. ChiNCs and RP@ChiNCs (1 μg/mL) were simultaneously treated with H_2_O_2_ (5 μM). Only the cell medium was used as the negative control, indicating inherent cellular ROS levels. The increased ROS levels upon H_2_O_2_ treatment were analyzed using H_2_DCFDA, which oxidizes to fluorescent 2′,7′-dichlorofluorescein. Fluorescence intensity was detected using a microplate reader at excitation and emission wavelengths of 485 and 535 nm, respectively.

The anti-inflammatory activity of RP@ChiNCs was evaluated based on its ability to inhibit NO production in LPS-stimulated RAW264.7 macrophage cells. LPS (100 ng/mL) was applied to the cells as an oxidative stress agent, followed by treatment with RP@ChiNCs (100 and 1000 μg/mL). The supernatant was collected after 24 h of incubation and reacted with the Griess reagent to detect NO. The absorbance was measured at 560 nm, and the NO concentration was calculated relative to the control group.

### 2.9. Collagen Synthesis of RP@ChiNCs

Human dermal fibroblasts were seeded into each well of a 48-well plate at a density of 2 × 10^4^ cells/mL. After 24 h of incubation at 37 °C and 5% CO_2_, RP@ChiNCs in DMEM (0.25%) were administered to the cells for 24 h. Then, the supernatant was collected to carry out the enzyme-linked immunosorbent assay (ELISA). The production of type I procollagen was analyzed using a Human Procollagen 1α1 Duoset ELISA kit (DY6220-05, R&D systems, Minneapolis, MN, USA), conforming to the manufacturer’s protocols. Collagen production after treatment with RP, RP in DMSO, or ChiNCs was evaluated as described above. The concentrations of RP and ChiNCs were equal to those of RP@ChiNCs.

### 2.10. In Vitro Wound Healing Activity of RP@ChiNCs

The wound-healing activities of ChiNCs and RP@ChiNCs were evaluated using the cell-scratch method. The experiment was performed under starvation conditions using cell medium without FBS. First, NIH 3T3 cells (150,000 cells/well) were seeded on a 24-well plate for 12 h in a CO_2_-humidified incubator at 37 °C for the formation of a fibroblast monolayer. A critical-size defect was created by scratching the cell layer using a sterile P1000 micropipette tip; the cells in the control groups did not proliferate in the defect within 24 h. Detached cell debris was washed twice with cell media. RP@ChiNCs (0.01, 0.1, and 1.0 mg/mL) were applied to the cells for different time periods (0, 4, 8, and 24 h). Wound closure was monitored during the 24 h of incubation under a DMi1 light microscope (Leica, Wetzlar, Germany), and the wound gap distance was calculated using ImageJ software 1.8.0 (NIH, Bethesda, MD, USA).

### 2.11. Statistical Analysis

All experiments were repeated at least three times (*n* = 3), and the resulting data were presented as mean ± standard deviation. The significance of differences in data was evaluated using Student’s *t*-test and one-way *ANOVA*. The *p*-values less than 0.05 were considered statistically significant.

## 3. Results

### 3.1. Preparation and Characterization of RP@ChiNCs

**Figure 1:**
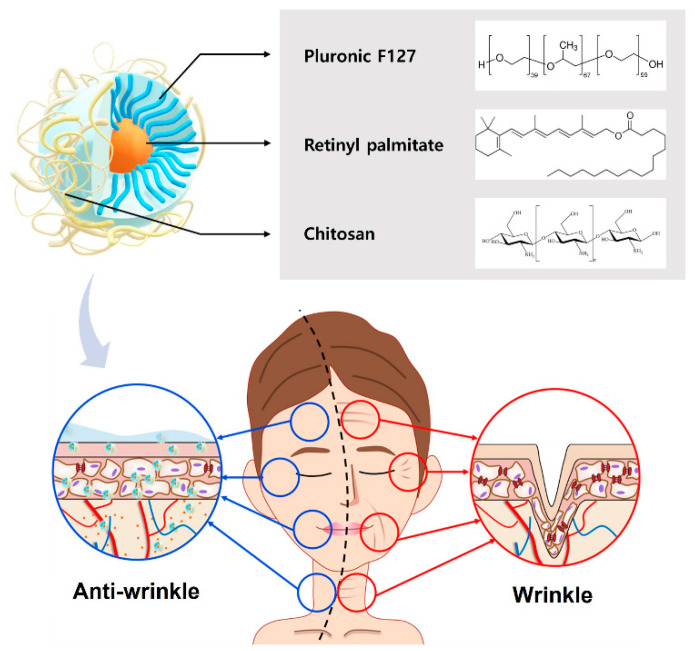
Schematic of the antiwrinkle efficacy of RP@ChiNCs containing Pluronic F127, retinyl palmitate, and chitosan.

**Figure 2:**
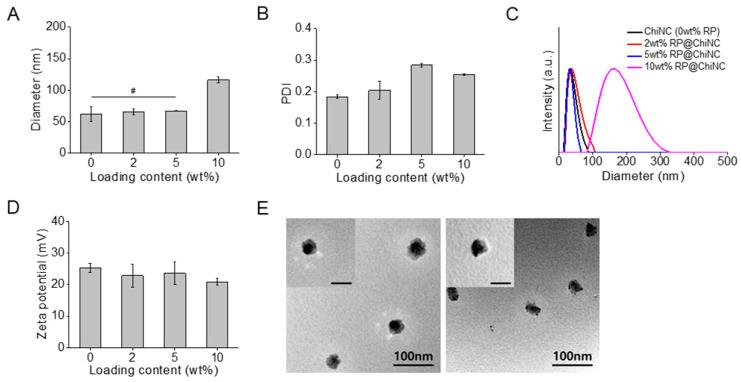
Characterization of RP@ChiNCs with different loading contents at 37 °C. (**A**) Hydrodynamic diameters, (**B**) polydispersity indices (PDI), (**C**) size distributions, and (**D**) zeta potentials of RP@ChiNCs. (**E**) Transmission electron microscopy images of ChiNCs (left) and RP@ChiNCs (5 wt.%, right). Inset scale bar: 50 nm. # *p* > 0.05.

Different amounts of hydrophobic RP were loaded into the ChiNCs using a nanoprecipitation method that has been widely used to prepare nanocarriers to improve the stability and retention rate of drugs [28]. As depicted in Figure 1, the antiwrinkle RP@ChiNCs comprise PF127, RP, and chitosan. Both Pluronic and RP were dissolved in water-miscible acetone and gradually mixed dropwise in water. A hydrophobic core of Pluronic encapsulated RP, and its hydrophilic shell provided steric hindrances. Furthermore, chitosan has been used as a transdermal penetration enhancer owing to its effect on keratin structure and cell membrane potential [29]. The optimal LC of RP was determined based on the physical properties of the RP@ChiNCs. The hydrodynamic diameters of RP@ChiNCs were maintained without significant changes up to an LC of 5 wt.%, as illustrated in Figure 2A (# *p* > 0.05). The diameter of bare ChiNCs was 62 ± 12 nm, and those after loading 2 and 5 wt.% RP were 65 ± 5 nm and 67 ± 1 nm, respectively. However, encapsulation of over 10 wt.% RP resulted in an increase in diameter to 116 ± 5 nm. This indicates the drug delivery capability of ChiNCs up to an LC of 5 wt.%. The PDIs of RP@ChiNCs were below 0.3 regardless of the LC, indicating that their sizes were uniformly distributed (Figure 2B). Accordingly, the size-distribution spectra of RP@ChiNCs corroborated the previous results (Figure 2C). Notably, the diameter of PluNCs was 18 ± 2 nm, while the encapsulation of RP increased the size of RP@PluNCs to 53 ± 2 nm (Supplementary Figure S1). Considering that the diameter of RP@ChiNCs was 62 nm, this indicates the presence of a chitosan layer on the surface of the PluNCs. The PDIs were all below 0.3, representing homogeneity in their sizes. The zeta potential of RP@ChiNCs indicated that they were positively charged, possibly because of the amine groups in the chitosan structure (Figure 2D). At a pH range of 5.0 to 6.0, amino groups on the chitosan tend to be protonated because their pKa values are approximately 6.5, facilitating the stabilization of the NCs. Considering that encapsulation of RP did not affect the zeta potential of the NCs, it was estimated that RP was present inside the NCs instead of being attached to their surface. Furthermore, the TEM images shown in Figure 2E indicate that both ChiNCs and RP@ChiNCs (5 wt.%) were similar in size and shape. Spherical morphologies of NCs were observed with an approximate size of 40 nm. The size discrepancy between DLS and TEM analyses was because of shrinkage of the NCs during sample preparation. This suggests that the optimal LC of RP in ChiNCs was 5 wt.%. Therefore, further experiments were performed using RP@ChiNCs (5 wt.%). The LC and LE of the optimized RP@ChiNCs were approximately 4.9 wt.% and 99.9%, respectively. In the study of Baghirova et al., RP was loaded in poly(lactic-co-glycolic acid) instead of Pluronic F127 [30]. However, Span 60 was additionally added for encapsulation, and the resulting nanoparticles had a larger particle size with lower encapsulation efficiency. In the current study, the diameters of the RP@ChiNCs were lower than 100 nm with much higher LE. This may facilitate the effective and efficient delivery of RP for cosmetic and biomedical uses.

### 3.2. Stability of RP@ChiNCs

**Figure 3:**
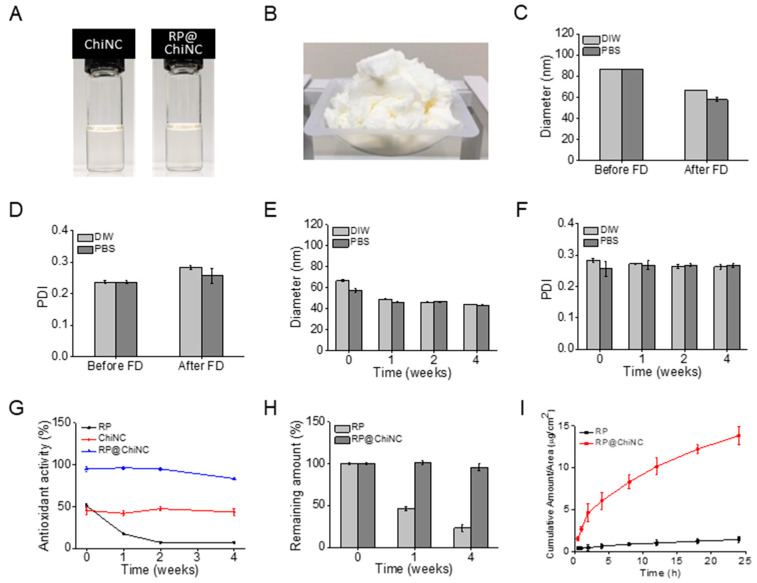
Stability analysis of RP@ChiNCs (5 wt.%) under different conditions. (**A**) Photographs of ChiNC and RP@ChiNC solutions. (**B**) Photograph of lyophilized RP@ChiNCs in the powder state. (**C**) Hydrodynamic diameters and (**D**) PDI of RP@ChiNCs in deionized (DI) water and phosphate-buffered saline (PBS) before and after freeze drying (FD). (**E**) Hydrodynamic diameters and (**F**) PDI of RP@ChiNCs after 4 weeks of storage in DI water and PBS at 37 °C and 100 rpm. Antioxidant activities of RP@ChiNCs (5 wt.%). (**G**) Results of DPPH radical scavenging assay of RP, ChiNCs, and RP@ChiNCs after 4 weeks of storage at 37 °C. (**H**) The percentage of non-degraded RP in RP@ChiNCs compared to bare RP at different storage time points at 37 °C. (**I**) In vitro skin permeation of RP and RP@ChiNCs at different time intervals in a Franz-type diffusion cell.

The aqueous solution of RP@ChiNCs in Figure 3A was easily lyophilized into a powder for ease of storage and transportation, as illustrated in Figure 3B. The diameter and PDI of freeze-dried RP@ChiNCs barely changed when they were redispersed in DI water and PBS (Figure 3C,D). This indicates that RP@ChiNCs can be stored as a powder before use without any challenges in its solubilization. Considering that several types of nanoparticles require cryoprotectants such as sucrose, glucose, mannitol, and trehalose for successful lyophilization without aggregation, RP@ChiNCs exhibited good lyophilization stability [31]. Further, the dispersion stability of RP@ChiNCs was analyzed for 4 weeks in DI water and PBS at 37 °C (Figure 3E,F). The hydrodynamic diameters and PDIs were maintained against aggregation, demonstrating the stable application of the NCs under physiological conditions.

### 3.3. In Situ Antioxidant Activity of RP@ChiNCs

The synergistic antioxidant activities of RP and ChiNCs were evaluated using a DPPH radical scavenging assay. RP is an effective antioxidant [32]. However, it is susceptible to degradation upon exposure to light and elevated temperatures [33]. Accordingly, the antioxidant activity of bare RP decreased from 51% to 7.4% during the 4 weeks of storage at 37 °C (Figure 3G). This was because RP was degraded to an inactive form, and a smaller amount of RP remained, as indicated in Figure 3H. Therefore, ChiNCs effectively protect bioactive RP and enhance its antioxidant activity. In a previous study, chitosan was demonstrated to be an effective delivery system for RP owing to its beneficial effect on the antioxidant activity of RP [18]. In particular, chitosan with a high DAC percentage and low MW was used to prepare ChiNCs, as it possessed the strongest antioxidant activity among other types of chitosan (Supplementary Figure S2). ChiNCs exhibited a stable antioxidant activity of approximately 45% over a sufficiently long period without degradation. Consequently, less than 5% of RP was degraded in the RP@ChiNCs after 4 weeks of incubation, and RP@ChiNCs had a remarkably high antioxidant activity of 95%.

### 3.4. pH Measurements and Skin Permeation of RP@ChiNCs

Appropriate pH values and skin penetration of RP@ChiNCs are vital for successful topical application. While the natural pH of the human skin ranges from 4–6, any change in skin acidity can cause skin irritation or bacterial infections [34]. The pH value of RP@ChiNC solution was in the range of 4.75−4.89, indicating its suitability for topical application. Further, it is noteworthy that weakly acidic drugs exhibit better skin permeation than basic drugs [35]. Therefore, it was considered that weakly acidic ChiNC solution may influence the ionization state of RP, thereby improving its penetration across the skin [36]. Hydrophobic RP typically exhibits low skin penetration [16]. It is a more stable form of retinol obtained via esterification; however, its lipophilicity limits its penetration through the phospholipid bilayers of the keratinocyte membrane [37]. As illustrated in Figure 3I, miniscule amounts of RP penetrated the skin membrane after 24 h. In contrast, RP@ChiNCs exhibited approximately 10-fold permeation. The beneficial role of ChiNCs in skin permeation was thoroughly discussed by Ma et al., who found that the chitosan disrupted the secondary structure of keratin, loosened the tight junctions of epidermal cells, and interacted with the intercellular lipids [38]. Therefore, chitosan has been used in several transdermal drug delivery systems. In another study, a hydrophobic immunomodulatory drug called imiquimod was loaded into ChiNCs to enhance their transdermal absorption and skin retention [39]. Bussio et al. achieved effective transcutaneous vaccination using ChiNCs for the transdermal delivery of ovalbumin [40]. This implies that ChiNCs are a potent skin-penetration enhancer of antioxidant and antiwrinkle RP.

### 3.5. In Vitro Cytotoxicity, Antioxidant, and Anti-Inflammatory Activity of RP@ChiNCs

**Figure 4:**
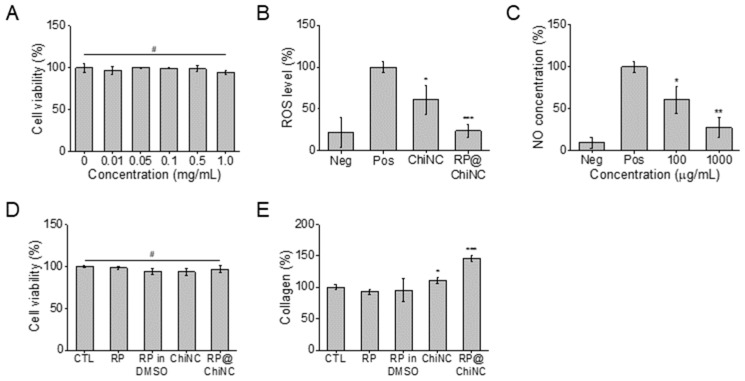
In vitro biocompatibility and efficacies of RP@ChiNCs (5 wt.%). (**A**) Cytotoxicity of RP@ChiNCs in NIH 3T3 fibroblast cells. (**B**) In vitro antioxidant activity and (**C**) in vitro anti-inflammatory efficacy of ChiNCs and RP@ChiNCs. Effects of RP on cell viability and collagen production in human dermal fibroblasts. (**D**) Cytotoxicity and (**E**) the amount of procollagen type 1 produced after 24 h treatment of RP, RP in DMSO, ChiNCs, and RP@ChiNCs. The concentration of RP was 0.25%. # *p* > 0.05, * *p* < 0.05, ** *p* < 0.01, and *** *p* < 0.001.

The viability of NIH 3T3 fibroblasts after treatment with RP@ChiNCs was measured to evaluate biocompatibility. Chitosan and Pluronic are biocompatible polymers [41,42]. Furthermore, RP is approved by the U.S. Food and Drug Administration for use in cosmetics, drugs, and food additives [43,44]. Accordingly, more than 90% of the cells survived treatment with RP@ChiNCs up to a concentration of 1 mg/mL (Figure 4A). The decrease in cell viability in the test groups was not significant compared to that in the control group (#*p* > 0.05). These results demonstrate the safety of RP@ChiNCs for biomedical applications. RP@ChiNCs did not cause any cytotoxicity. Furthermore, they were beneficial for ROS scavenging in H_2_O_2_-stimulated cells. As depicted in Figure 4B, H_2_O_2_ stimulation increased the normal intracellular ROS levels from 21.5% in the negative control group to 100% in the positive control group. The antioxidant ChiNCs and RP@ChiNCs decreased the ROS levels by 60.7% and 23.8%, respectively. Notably, the in vitro radical scavenging ability of ChiNCs improved after RP encapsulation, owing to the synergistic antioxidant activity of RP and chitosan. This indicates that ChiNCs not only provide a stabilizing effect as a drug delivery system but also enhance the efficacy of RP@ChiNCs. Similarly, LPS was used to induce excessive NO production in RAW264.7 macrophage cells. As illustrated in Figure 4C, RP@ChiNCs reduced the NO concentration from 100% to 60.6% to 27.3% in a dose-dependent manner. Considering that NO overproduction is a major cause of severe inflammation, RP@ChiNCs were determined to have high anti-inflammatory efficacy.

### 3.6. In Vitro Wound Healing Efficacy and Collagen Synthesis of RP@ChiNCs

**Figure 5:**
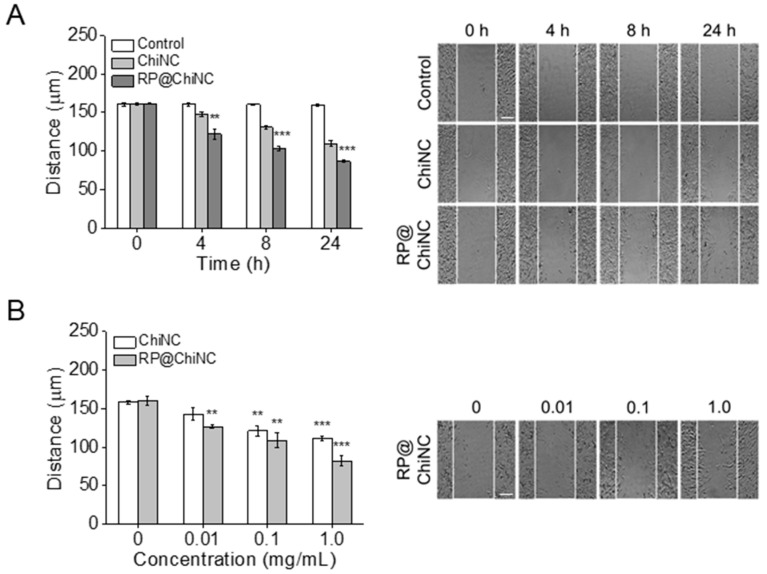
In vitro wound healing efficacies of ChiNCs and RP@ChiNCs (5 wt.%). (**A**) Wound gap distances (right) and cell morphologies (left) after 24 h of treatment with ChiNCs and RP@ChiNCs at different incubation intervals (1 mg/mL). (**B**) Wound closure after 24 h of treatment with RP@ChiNCs at various concentrations. Scale bar = 50 μm. ** *p* < 0.01 and *** *p* < 0.001.

RP is a potent antiwrinkle agent owing to its strong antioxidant and anti-inflammatory effects [45]. Because RP@ChiNCs exhibited enhanced efficacy in situ and in vitro, their collagen production and wound-healing activity were considerably more prominent than those of RP or ChiNCs alone. Collagen production by RP@ChiNCs in human dermal fibroblasts was analyzed as a measure of their antiwrinkle ability (Figure 4D,E). Due to their biocompatibility, RP, ChiNCs, and RP@ChiNCs were not cytotoxic in human dermal fibroblasts, and the amount of procollagen type 1 produced after 24 h of treatment was compared. Both RP and RP in DMSO hardly produced any intracellular collagen. ChiNCs exhibited a better collagen production of approximately 11.1%. This may be explained by the enhanced cell penetration in the presence of chitosan. Accordingly, RP@ChiNCs produced a large amount of collagen (46.5%) owing to the synergistic effect of RP and chitosan, indicating their prominent antiwrinkle ability. Collagen plays a crucial role in maintaining skin elasticity and delaying skin aging [46]. Therefore, the amount of collagen produced can be an indication of the anti-aging activity of RP@ChiNCs for cosmetic and biomedical applications. RP@ChiNCs promoted cell proliferation and migration throughout the wound area over time (Figure 5A). While the wound closure in the control group was negligible, it narrowed from 160 to 110 and 86 μm with ChiNCs and RP@ChiNCs, respectively, after 24 h of treatment. Notably, the wound-healing efficacies of ChiNCs and RP@ChiNCs depended on the nanocapsule concentration. Therefore, the wound closure was faster at higher concentrations, as shown in Figure 5B. This result correlates with previous studies that demonstrated the beneficial role of RP and chitosan in wound repair. Baghirova et al. reported that RP-loaded nanoparticles increase keratinocyte cell proliferation [30]. Furthermore, RP promotes fibroblast cell turnover, diminishes collagen loss, and alleviates inflammation in injured skin. The effect of chitosan on fibroblast proliferation was extensively investigated in a study by Howling et al., who demonstrated that chitosan stimulates cell proliferation by interacting with serum components or acting as a progression factor [47].

## 4. Conclusions

ChiNCs were developed as nanocarriers of hydrophobic RP for enhanced stability and efficacy. RP@ChiNCs could load up to 5 wt.% RP without significant changes in their characteristics. The encapsulated RP insignificantly degraded during the 4 weeks of storage at 37 °C, whereas the bare RP disintegrated rapidly. Therefore, the RP@ChiNCs possess high DPPH radical-scavenging activity, regardless of the storage time. Furthermore, ChiNCs are effective skin-penetration enhancers, resulting in a 10-fold improvement in skin permeability in RP@ChiNCs compared to that of lipophilic RP. In in vitro assays, biocompatible RP@ChiNCs effectively inhibited ROS and NO production in fibroblasts and macrophages stimulated with oxidative stress agents. Consequently, they favored collagen production and wound healing owing to their antioxidant and anti-inflammatory effects. This indicates that RP@ChiNCs are a promising nanoplatform for the delivery of hydrophobic and unstable RP for applications in antiwrinkle cosmetics, drugs, and healthcare foods. Future research may focus on the development of functional cosmetics for wrinkle improvement, while optimization of the preparation of NCs is required to load different types of drugs on a large scale.

## Data Availability

The data presented in this study are available within the article and its Supplementary Material.

## Supplementary Materials

The following supporting information can be downloaded at: https://www.mdpi.com/article/10.3390/antiox12111913/s1, Figure S1: Characterization of PluNCs, RP@PluNCs, and RP@ChiNCs. (A) Hydrodynamic diameters and (B) PDI of PluNCs, RP@PluNCs, and RP@ChiNCs; Figure S2: DPPH radical scavenging activities of 12 types of chitosan with different percentages of DAC and MWs.

## Abbreviations

CCK8: Cell Counting Kit-8; ChiNCs, chitosan-coated nanocapsules; DAC, deacetylation; DCP_mod_-SLNs, surface-modified lipid nanoparticles using dicetyl phosphate; DI, deionized; DLS, dynamic light scattering; DMEM, Dulbecco’s modified Eagle’s medium; DMSO, dimethyl sulfoxide; DPPH, 2,2-diphenyl 1-1-picrylhydrazyl; ELISA, enzyme-linked immunosorbent assay; H_2_DCFDA, 2′,7′-dichlorodihydrofluorescein diacetate; FBS, fetal bovine serum; FD, freeze drying; HPLC, high-performance liquid chromatography; LC, loading content; LE, loading efficiency; LPS, lipopolysaccharides from *E. coli* O111:B4; MTT, 3-(4,5-dimethythiazol-2-yl)-2,5-diphenyl tetrazolium; MW, molecular weight; NCs, nanocapsules; PBS, phosphate-buffered saline; PDI, polydispersity index; PF127, Pluronic F127; PluNCs, Pluronic nanocapsules; ROS, reactive oxygen species; RP, retinyl palmitate; RP@ChiNC, RP-loaded chitosan-coated nanocapsules; SLNs, solid lipid nanoparticles; TEM, transmission electron microscopy.
