# Supplementary material for: Super-Antioxidant Vitamin A Derivatives with Improved Stability and Efficacy Using Skin-Permeable Chitosan Nanocapsules
Source: Antioxidants (Basel). 2023 Oct 26;12(11):1913. doi: 10.3390/antiox12111913 (PMC10669859; doi:10.3390/antiox12111913)
Supplement: Supplementary file 1 [file antioxidants-12-01913-s001.zip › antioxidants-2639701-supplementary.pdf]

Supporting Information

Super-Antioxidant Vitamin A Derivatives with Improved Stability and Efficacy Using Skin-Permeable Chitosan Nanocapsules

Hyeryeon Oh [1,2,†], Jin Sil Lee [1,2,†], Sunghyun Kim [1], Jeung-Hoon Lee [3], Yong Chul Shin [3,4] and Won Il Choi [1,*]

[1] Center for Bio-Healthcare Materials, Bio-Convergence Materials R&D Division, Korea Institute of Ceramic Engineering and Technology, 202, Osongsaengmyeong 1-ro, Cheongju 28160, Republic of Korea; hyeryeon.oh@kicet.re.kr (H.O.); jslee92@kicet.re.kr (J.S.L.); shkim0519@kicet.re.kr (S.K.)
[2] School of Materials Science and Engineering, Gwangju Institute of Science and Technology, 123, Cheomdan-gwagiro, Gwangju 61005, Republic of Korea
[3] SKINMED Co., Ltd., Daejeon 34028, Republic of Korea; jhoon@cnu.ac.kr (J.-H.L.); ycshin@amicogen.com (Y.C.S.)
[4] Amicogen Inc., 64 Dongburo, 1259, Jinju 52621, Republic of Korea
* Correspondence: choi830509@kicet.re.kr; Tel.: +82-43-913-1513
† These authors contributed equally to this work.

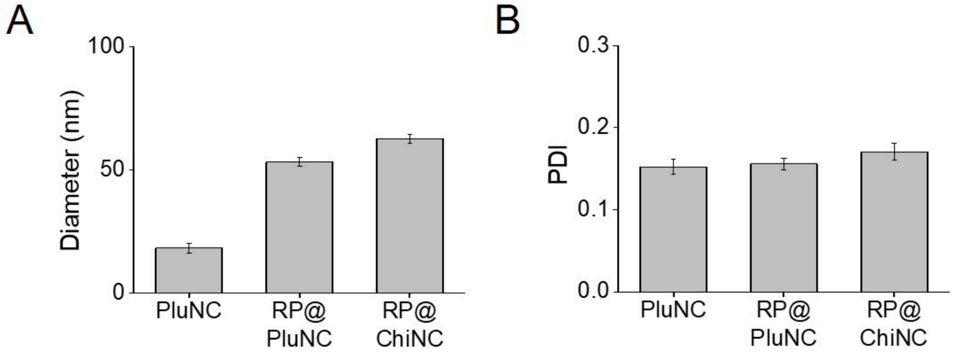

Figure S1. Characterization of PluNC, RP@PluNC, and RP@ChiNC. (A) Hydrodynamic diameters and (B) polydispersity indices (PDI) of PluNC, RP@PluNC, and RP@ChiNC.

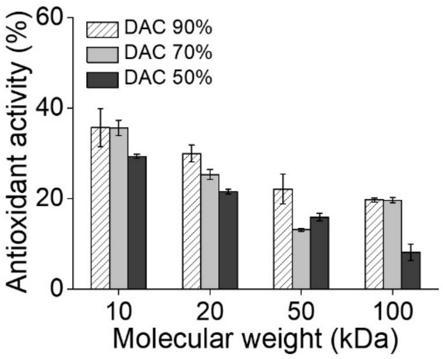

Figure S2. DPPH radical scavenging activities of 12 types of chitosan with different percentages of deacetylation and molecular weights.
